# Knowledge and Attitude Regarding Child Abuse Among Primary Healthcare Physicians and Interns in Al Qassim, Saudi Arabia

**DOI:** 10.7759/cureus.12270

**Published:** 2020-12-25

**Authors:** Abdulrhman Aldukhayel, Emad Aljarbou, Fatima M Alturki, Nouf S Almazyad, Ohud M Alsaqer, Raghad Almutairi

**Affiliations:** 1 Department of Family and Community Medicine, Qassim University, College of Medicine, Buraidah, SAU; 2 Medicine, Qassim University, College of Medicine, Buraidah, SAU

**Keywords:** neglect, child abuse, attitude, knowledge

## Abstract

Introduction

Child abuse can include physical abuse, psychological abuse, sexual assault, neglect, or failure to meet the child's basic needs. It can lead to major psychosocial problems resulting in long-lasting consequences for the child.

Objective

This study aimed to assess the knowledge and attitude (KA) of primary healthcare physicians and interns regarding child abuse in Al Qassim, Saudi Arabia.

Materials and methods

This multicenter, cross-sectional study was conducted among primary healthcare physicians and interns in the Qassim region, Saudi Arabia, from July to October 2020. A self-administered questionnaire was distributed among the targeted physicians using either an online platform or face-to-face interviews. The questionnaire was devised from the pre-tested literature and formal discussions with experts. A total of 292 primary healthcare physicians and interns participated in our study. A consent form and brief details of the study were given ahead of the recruitment. Data were tabulated in a Microsoft Excel spreadsheet (Microsoft Corporation, Redmond, WA); a separate codebook was prepared with a description of the variables and corresponding codes, and all statistical analyses were performed using IBM SPSS Statistics for Windows, Version 21.0 (IBM Corp., Armonk, NY).

Results

A total of 292 respondents were involved in the study. Among them, 45% (n=131) were medical interns, 39.4% (n=115) were residents, and the rest were either specialists (n=34; 11.6%) or consultants (n=12; 4.1%). Among the respondents, the KA level was found to be moderate, high, and low in 68.8%, 28.4%, and 2.7%, respectively. The overall mean KA score was 81.1 [standard deviation (SD): 9.56] out of a possible 115 points. Factors associated with high KA were age (>30 years), non-Saudi nationality, having children, and having studied outside Saudi Arabia; The only factor significantly associated with low levels of KA was being a pediatrician (p<0.001).

Conclusion

Overall, the participants displayed adequate levels of KA regarding child abuse. Better KA was observed among expatriate physicians who were parents themselves and had earned their degrees outside the Kingdom of Saudi Arabia. However, pediatricians demonstrated poor knowledge with regard to child abuse.

## Introduction

Child abuse encompasses physical abuse, psychological abuse, sexual assault, and/or neglect and failure to meet the child's basic needs. It can lead to major social problems resulting in long-lasting consequences for the child [1]. However, cultural differences around the world concerning child discipline and parenting comprise a wide range of approaches making a clear, singular definition of child abuse or neglect challenging to establish [2]. In 1990, the first case of child abuse was officially recorded in Saudi Arabia, and in 1994, a government committee was formed to oversee the protection of children’s rights in the country [3]. Physicians are obligated to report cases of child abuse to the concerned social welfare organizations or government authorities to provide early interventions for abuse victims to prevent further abuse [4]. Physicians can use screening instruments for assistance in recognizing child abuse at an early stage, thereby helping to prevent any further harm [5]. Despite the obligation of reporting suspected cases of child abuse to child protective services, a 2015 Swedish study reported that more than 50% of pediatricians and general practitioners did not report cases of child abuse [6]. Moreover, 60% of general practitioners were not familiar with child abuse guidelines or local protocols within their clinics for handling such situations [6]. A 2017 study in Turkey reported that only 13% of physicians in their study received training on reporting child abuse [7]. Most physicians in that study (72%) knew of existing laws to protect children from abuse; however, the authors found that physicians did not report suspected child abuse cases due to a lack of knowledge about the procedures and a desire to avoid involvement in legal procedures [7]. A 2019 study in Saudi Arabia that included all physicians in the Abha region found that there was sufficient awareness regarding signs of suspected cases of child abuse among physicians, but cases were still underreported due to a lack of knowledge about the reporting process and also due to community barriers [8].

In light of this, we conducted this study to assess the knowledge and attitude (KA) among primary healthcare physicians and interns regarding child abuse in Al Qassim, Saudi Arabia.

## Materials and methods

We conducted a multicenter, cross-sectional study among primary healthcare physicians and interns in the Qassim region of Saudi Arabia from July to October 2020. A self-administered questionnaire was distributed among the targeted physicians using either an online platform or via face-to-face interviews. The questionnaire was devised from the literature and with input from experts via formal discussions. A total of 292 primary healthcare physicians and interns participated in our study. Participants provided written informed consent before inclusion in the study. Responses were collected and tabulated in a Microsoft Excel spreadsheet (Microsoft Corporation, Redmond, WA).

Scoring 

The criteria for KA regarding child abuse were encapsulated in 23 questions (Table 1) with the provision to provide answers through a 5-point Likert scale, with answer options ranging from "strongly disagree" coded as 1 to "strongly agree" coded as 5. The questionnaire consisted of four domains: knowledge about child abuse and neglect (CAN; eight questions), reasons why child abuse is underreported in Saudi Arabia (five questions), acts of abuse toward children (five questions), and acts of child neglect (five questions). The total KA score was calculated by summing up the answers to all 23 questions, with a possible score range of 23-115, with higher scores indicating higher levels of KA regarding child abuse. Using 50% and 75% of the total score points to determine the level of KA, participants were classified as having low KA if the score was from 23 to 58 points; scores of 59 to 86 were considered as moderate KA, and scores of 87 to 115 were classified as high KA.

**Table 1:** Assessment of knowledge and attitude regarding child abuse and neglect (n=292) CAN: child abuse and neglect; SD: standard deviation

Statement		Score, mean ± SD (out of 5)	Agreed, n (%)
Knowledge about CAN			
1.	I have enough training to deal with CAN	2.85 ± 1.07	78 (26.7%)
2.	We need to redefine CAN in Saudi Arabia according to our culture and religion	3.74 ± 1.22	199 (68.2%)
3.	The present supportive services to deal with CAN in Saudi Arabia are adequate	2.78 ± 1.04	60 (20.5%)
4.	I prefer resolving a case of child abuse myself rather than reporting it to the police	2.25 ± 1.18	53 (18.2%)
5.	I am aware of the reporting procedures regarding child abuse in my hospital in Saudi Arabia	2.85 ± 1.16	88 (30.1%)
6.	I am willing to report all suspected child abuse cases	3.94 ± 1.04	204 (69.9%)
7.	I prefer limiting my reporting of child abuse to life-threatening injuries	2.24 ± 1.27	61 (20.9%)
8.	Child abuse is underreported in Saudi Arabia	3.56 ± 1.11	156 (53.4%)
Reasons why child abuse is underreported in Saudi Arabia			
9.	It is not legally mandatory to report child abuse	2.71 ± 1.11	81 (27.7%)
10.	Reporting might not be good for the sake of the child	3.21 ± 1.38	136 (46.6%)
11.	Reporting procedures are unclear	3.46 ± 1.11	149 (51.0%)
12.	Reporting child abuse to authorities is not yet acceptable in our community	3.50 ± 1.10	162 (55.5%)
13.	Fear of parent response	3.52 ± 1.07	162 (55.5%)
Acts of abuse toward children			
14.	Burning the child for misbehavior	4.11 ± 0.95	212 (72.6%)
15.	Locking the child alone at home	3.90 ± 0.97	205 (70.2%)
16.	Severe beating that leaves marks on the child's body	4.16 ± 0.93	217 (74.3%)
17.	Parents throwing different objects at the child when angry	4.00 ± 0.98	211 (72.3%)
18.	Parents smoking in the presence of the child	4.01 ± 0.85	220 (75.3%)
Acts of child neglect			
19.	Parents refusing to send the child to school	4.12 ± 0.97	219 (75.0%)
20.	Parents refusing medical treatment or surgical intervention necessary for their child	4.08 ± 0.94	217 (74.3%)
21.	Child with severe dental problems, which are not treated	4.02 ± 0.88	222 (76.0%)
22.	Parents paying no attention to the child's cleanness	3.98 ± 0.91	217 (74.3%)
23.	Child failing to thrive due to social deprivation	4.07 ± 0.90	217 (74.3%)

Statistical analysis

Descriptive statistics were demonstrated using numbers, percentages, mean, and standard deviation (SD), as appropriate. Between comparisons, the Mann-Whitney U test and the Kruskal-Wallis test were applied. A p-value of <0.05 was considered statistically significant. Normality tests were conducted using the Shapiro-Wilk test. All statistical analyses were carried out using IBM SPSS Statistics for Windows, Version 21.0 (IBM Corp., Armonk, NY).

## Results

This cross-sectional study enrolled 292 primary healthcare physicians and interns across the Qassim region. Table 2 presents the sociodemographic characteristics of the respondents. Most respondents belonged to the age group of 22-30 years (64.3%); more than half (56.5%) of them were men, and approximately two-thirds (66.1%) were Saudis. Regarding their professional status, 44.9% were medical interns, and 39.4% were residents. Furthermore, 40.1% were married, with approximately two-thirds (64.7%) having no children. With regard to specialty, nearly 45% were internists, while 21.2% were general practitioners. Additionally, nearly two-thirds (65.1%) of the respondents had received their education within Saudi Arabia, with 63.7% having practiced medicine for fewer than five years.

**Table 2:** Sociodemographic characteristics of physicians (n=292)

Study variables	N (%)
Age group	
22–30 years	188 (64.3%)
31–40 years	42 (14.4%)
41–50 years	39 (13.4%)
>50 years	23 (07.9%)
Gender	
Male	165 (56.5%)
Female	127 (43.5%)
Nationality	
Saudi	193 (66.1%)
Non-Saudi	99 (33.9%)
Professional level	
Medical Intern	131 (44.9%)
Resident	115 (39.4%)
Consultant	12 (04.1%)
Specialist	34 (11.6%)
Married	
Yes	117 (40.1%)
No	175 (59.9%)
Children	
None	189 (64.7%)
1–2	35 (12.0%)
>2	68 (23.3%)
Specialty	
General practitioner	62 (21.2%)
Family medicine	48 (16.4%)
Pediatrics	25 (08.6%)
Internist	131 (44.9%)
Other	26 (08.9%)
Country of medical education	
Saudi Arabia	190 (65.1%)
Other Arab countries	84 (28.8%)
European country	03 (01.0%)
Others	15 (05.1%)
Years in practice	
<5 years	186 (63.7%)
5–10 years	36 (12.3%)
>10 years	70 (24.0%)

KA was divided into four domains: knowledge about CAN, reasons why child abuse is underreported in Saudi Arabia, acts of abuse toward children, and acts of child neglect. The score ascribed to answer options ranged from 1 to 5 points, where a score of five indicated the highest level of awareness. Firstly, regarding knowledge about CAN, respondents showed good knowledge with respect to the following statements: "I am willing to report all suspected child abuse cases" (mean: 3.94), "We need to redefine CAN in Saudi Arabia according to our culture and religion" (mean: 3.74), and "Child abuse is underreported in Saudi Arabia" (mean: 3.56). Respondents' knowledge was low with regard to the following statements: "I prefer limiting my reporting of child abuse to life-threatening injuries" (mean: 2.24) and "I prefer resolving a case of child abuse myself rather than reporting it to police" (mean: 2.25). Secondly, regarding the category of reasons why child abuse is underreported in Saudi Arabia, respondents showed good KA with respect to the following sentences: "Fear of parent response" (mean: 3.52) and "Reporting child abuse to authorities is not yet acceptable in our community" (mean: 3.50). KA was low regarding the statement "It is not legally mandatory to report the child abuse" (mean: 2.71). Thirdly, for the acts of abuse toward children, respondents showed high KA regarding most of the statements, especially with respect to "Severe beating that leaves marks on the child body" (mean: 4.16) and "Burning the child for misbehavior" (mean: 4.11). Finally, related to the acts of child neglect, respondents also showed high KA with respect to most of the statements, and it was really noteworthy pertaining to the statements "Parents refusing to send the child to school" (mean: 4.12) followed by "Parents refusing medical treatment or surgical intervention necessary for their child" (mean: 4.08) and "Child failing to thrive due to social deprivation" (mean: 4.07).

Table 3 summarizes the descriptive statistics of the KA regarding CAN, including the mean score of knowledge about CAN (24.2), reasons why child abuse is underreported (16.4), acts of abuse toward children (20.2), and acts of child neglect (20.3). The overall mean score was 81.1 (SD: 9.56).

Based on the given criteria, we found a low level of CAN KA in 2.7% of respondents, a moderate level of KA in 68.8%, and a high level of KA in 28.4% of respondents.

**Table 3:** Descriptive statistics of the knowledge and attitude regarding CAN (n=292) CAN: child abuse and neglect; SD: standard deviation

Variables	Number of items	Mean ± SD
Knowledge about CAN	08	24.2 ± 4.27
Reasons why child abuse is underreported in Saudi Arabia	05	16.4 ± 3.98
Acts of abuse toward children	05	20.2 ± 3.81
Acts of child neglect	05	20.3 ± 3.97
Total score	23	81.1 ± 9.56

When measuring the differences between the KA scores in relation to physician sociodemographics, the KA of those in the older age group (>30 years; T=-2.723; p=0.025), non-Saudis (T=-3.451; p=0.002), those who have children (T=2.465; p=0.031), and those who had earned their degree outside of Saudi Arabia (T=-2.911; p=0.005) was significantly higher than their counterparts. Those in pediatric specialty showed significantly lower KA scores compared to those in other specialties (F8.274; p<0.001; Table 4). On the other hand, gender, professional level, marital status, and years in practice did not significantly affect the KA score (all p>0.05).

## Discussion

CAN is documented in every country and culture, and child maltreatment is associated with several consequences, including parental depression, stress, and social isolation [9]. The protection of children is essential, and suspected child abuse cases should be reported and documented to the concerned authorities who can assess the situation and help the young ones [9]. Appropriate investigation and intervention to help the abuse victims are necessary to alleviate the trauma of these children and their families. The purpose of the present study was to determine the KA among primary healthcare physicians regarding child abuse.

Unfortunately, there appear to be gaps in the system that allows for underreporting of abuse cases, and the knowledge levels are not consistent: several studies have reported adequate knowledge regarding child abuse among physicians [8,10-13], while other studies report insufficient knowledge levels [14,15]. A long-term study investigating potential child abuse among all children younger than 18 years of age showed that the annual prevalence of child abuse is 1%, with emotional neglect being the most common type [16]. A study in France in 2015 on the level of knowledge about diagnosing child abuse among family physicians showed that 30% had previous training for dealing with abuse cases [17]. In that study, 82% of physicians reported having suspected cases of child abuse. The study showed that the main obstacles to reporting such cases were the fear of misdiagnosis and the fear of separating the children from their families with a lack of knowledge about the reporting process [8]. Sathiadas et al. [18] reported that although healthcare professionals demonstrated good overall knowledge, 65.8% of physicians were unsatisfied with their knowledge and expressed the desire for some form of education on child maltreatment to further their understanding. AlBakr et al. [16] reported that the overall knowledge among all primary healthcare physicians in Al-Khobar City in the Eastern Region of Saudi Arabia about child abuse was significantly higher among those aged 36-40 years, which aligned with our findings related to the age of the respondents. They also reported that longer experience was associated with higher levels of knowledge [16]; however, we did not find this to be true in our study as we found no significant association between the years in practice and the overall KA score.

We found that those who had earned their degree outside Saudi Arabia had better KA scores than those who studied within the Kingdom, This contrasts with the findings of Habib [13], who found that respondents receiving their medical education in Saudi Arabia scored statistically significantly higher in CAN knowledge regarding "professional experience about child abuse and negligence" compared to pediatricians who completed their medical education in Western countries. Furthermore, Habib [13] indicated that gender differences were not significant, which was consistent with our results.

In Iran, investigators have reported that pedodontists had higher levels of knowledge about CAN than general dentists [12]. This did not seem to disagree with our results, as we observed that specialty significantly influences the knowledge score, with pediatricians exhibiting lower knowledge scores than doctors in other specialties. However, our results contradict the report by Sathiadas et al., who found that the knowledge, attitude, and behavior of healthcare professionals toward child abuse were significantly good among respondents within the pediatric specialty [18]. Similarly, we also noted that physicians with children have significantly better KA scores than those without children. 

Moreover, it is essential to mention that more than 53% of the physicians agreed that child abuse was underreported within Saudi Arabia. This is consistent with the findings of Alsaleem et al., who reported that 64% of primary healthcare physicians noticed underreported child abuse [10]. Alnasser et al. emphasized that child maltreatment in Saudi Arabia was common [12]. Several factors have been attributed to the underreporting of child abuse. In our study, the most commonly mentioned reason for underreported instances of child maltreatment was fear of parent response followed by the feeling that reporting of child abuse is not yet acceptable and reporting procedures are not clear, which was also in accordance with the findings of Alsaleem et al. [10]. Also, approximately 70% of physicians thought it is important to redefine CAN in Saudi Arabia according to the country's culture and religion, which was strikingly similar to the findings of Alsaleem et al. [10].

There are several limitations to this study. Specifically, the study was conducted in a single region in Saudi Arabia. Therefore, the results may not be generalizable, replicable, or even applicable to other regions. This implies that the convenience sampling method has the potential for sampling bias. Additionally, this study design may have introduced some recall bias and subjectivity.

## Conclusions

Most of the participants in this study showed adequate KA regarding CAN. High KA scores were seen among expatriate physicians who were parents and older than 30 years in age. Pediatricians demonstrated poor KA with regard to CAN. It is necessary to address the gaps in KA regarding CAN, most especially among pediatricians. Given that pediatricians are primarily in charge of children's health and wellness, they need to have adequate knowledge and information about CAN. More CAN courses and training should be implemented to address the lack of KA regarding CAN among pediatricians.

## Appendices

Knowledge and Attitude regarding child abuse among primary healthcare physician in Qassim, Saudi Arabia

This questionnaire aims to assess your knowledge and attitude regarding child abuse. its results will help us to improve this aspect.

1. Gender

 male

 female

2. Age: ...............

3. Married

 Yes

 No

4. No. of children: …………….

5. Nationality 

 Saudi

 Non-Saudi

6. Education level

 Resident

 Specialist

 Consultant

 Other …………………..

7. Specialty

 GP

 Family medicine

 Pediatrics

 Internist

 Other……………………

8. Country of medical education

 Saudi Arabia

 Arab country

 European country

 North America

 Other ……………………

9.Years of practice: ………………

**Table 4:** Child abuse and neglect scoring system

Kindly score the fowling items regarding child abuse and neglect out of 5 as follows: Strongly disagree: 1, Disagree: 2, Neutral: 3, Agree: 4, Strongly agree: 5	Score
I have enough training to deal with child abuse and neglect	
We need to redefine child abuse and neglect in Saudi Arabia according to our culture and religion	
The present supportive services to deal with child abuse and neglect in Saudi Arabia are adequate	
I prefer resolving a case of child abuse myself rather than reporting it to the police	
I am aware of the reporting procedures of child abuse in my hospital in Saudi Arabia	
I am willing to report all suspected child abuse cases	
I prefer limiting my reporting of child abuse to life-threatening injuries	
Child abuse is underreported in Saudi Arabia	
The following might be the reasons why child abuse is underreported in Saudi Arabia:	Score
It is not legally mandatory to report child abuse	
Reporting might not be good for the sake of the child	
Reporting procedures are unclear	
Reporting child abuse to authorities is not yet acceptable in our community	
Fear of parent response	
Do you consider the following acts as abusive toward children?	Score
Burning the child for misbehavior	
Locking the child alone at home	
Severe beating that leaves marks on the child's body	
Parents throwing different objects at the child when angry	
Parents who smoke in the presence of the child	
Do you consider the following acts as acts of child neglect?	Score
Parents refusing to send the child to school	
Parents refusing medical treatment or surgical intervention necessary for their child	
Child with severe dental problems, which are not treated	
Parents paying no attention to the child's cleanness	
Child failing to thrive due to social deprivation	
